# Bilateral non-bifurcating carotid arteries in a patient with recurrent cerebrovascular events

**DOI:** 10.1186/s42466-021-00154-9

**Published:** 2021-10-18

**Authors:** Lina Palaiodimou, Georgia Papagiannopoulou, Aikaterini Theodorou, Eleni Bakola, Maria Chondrogianni, Maria-Ioanna Stefanou, Elizabeth Andreadou, Stefanos Lachanis, Georgios Tsivgoulis

**Affiliations:** 1 Second Department of Neurology, National and Kapodistrian University of Athens, School of Medicine, “Attikon” University Hospital, Rimini 1, Chaidari, 12462 Athens, Greece; 2 First Department of Neurology, National and Kapodistiran University of Athens, School of Medicine, “Eginition” University Hospital, Athens, Greece; 3 Iatropolis Magnetic Resonance Diagnostic Centre, Athens, Greece; 4 Department of Neurology, The University of Tennessee Health Science Center, Memphis, TN USA

**Keywords:** Transient ischemic attack, Bilateral non-bifurcating carotid arteries, Carotid artery variation, MRI, MRA

## Abstract

**Introduction:**

Among congenital anomalies of the carotid artery circulation, the presence of a non-bifurcating carotid artery is extremely rare. Relevant cases with unilateral non-bifurcating carotid artery have scarcely been described in the literature. After extensive literature review, only one case with asymptomatic bilateral non-bifurcating carotid arteries associated with persistent proatlantal artery was identified.

**Methods:**

We present the case of a 40-year-old man with recurrent cerebrovascular events presenting non-bifurcating carotid arteries bilaterally.

**Results:**

A 40-year-old man presented in the emergency department with a transient ischemic attack. Past medical history included prior ischemic stroke of unknown etiology in the distribution of the left middle cerebral artery, untreated hyperlipidemia and tobacco use. Complete work-up in order to identify the underlying mechanism of the patient’s recurrent cerebrovascular events was negative, except for the finding of non-bifurcating carotid arteries bilaterally, associated with an extensive intracranial anastomosing arterial network. Long-term antiplatelet therapy and statins were administered as secondary stroke prevention therapy.

**Discussion:**

Previous reports suggest that non-bifurcating carotid arteries may be associated with atherosclerotic plaque formation in symptomatic cases due to shear stress, tortuosity or other local factors. However, in the absence of atherosclerosis, the pathogenic association of bilateral non-bifurcating carotid arteries with cerebrovascular events remains questionable, but may be considered when other stroke etiologies are excluded.

**Supplementary Information:**

The online version contains supplementary material available at 10.1186/s42466-021-00154-9.

## Introduction

Among congenital anomalies of the carotid artery circulation, the presence of non-bifurcating carotid artery is rare. Relevant cases with unilateral non-bifurcating carotid artery have scarcely been described in the literature (Additional file 1: Table S1). After extensive literature review, only one case with asymptomatic bilateral non-bifurcating carotid arteries associated with persistent proatlantal artery of the type I variety, anastomosing the right ICA and the right vertebral artery, was identified [1].

## Methods

We present the case of a 40-year-old man with recurrent cerebrovascular events presenting with non-bifurcating carotid arteries bilaterally.

## Case report

A 40-year-old Asian man presented in the emergency department with acute right-arm weakness and dysarthria, that spontaneously resolved within 18 h. The patient was asymptomatic and hemodynamically stable upon admission. Past medical history included prior ischemic stroke in the distribution of the left middle cerebral artery, for which the etiologic investigation, follow-up and treatment were inadequately conducted at an outside Institution, untreated hyperlipidemia with low-density lipoprotein levels of 160 mg/dl and tobacco use.

During the initial patient’s evaluation, an emergent brain CT was performed that excluded any acute brain lesions. Cervical duplex ultrasound revealed normal blood flow in the common carotid arteries and internal carotid arteries (ICA) with no evidence of carotid stenosis or atherosclerotic plaques. However, the carotid bifurcation and the external carotid arteries (ECA) could not be detected bilaterally. Transcranial color-coded duplex sonography was also performed, revealing normal blood flow in the large intracerebral arteries. Pulsatility index measured at the middle cerebral arteries bilaterally was within normal limits, indicating normal cerebral hemodynamics. No microembolic signals were identified after administration of agitated saline on Transcranial Doppler bubble study excluding the presence of a right-to-left shunt.

**Fig. 1:**
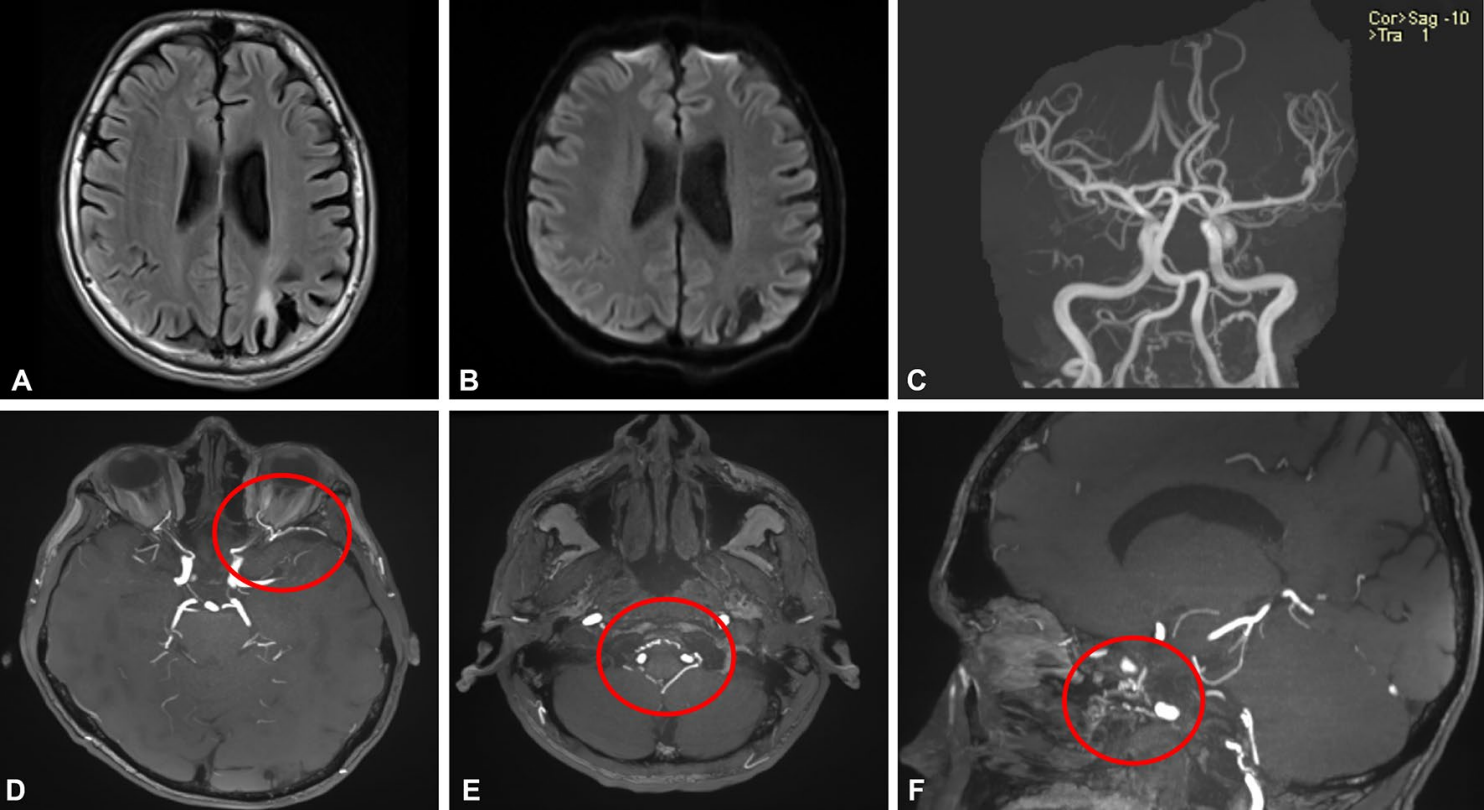
Brain MRI/MRA of a patient with recurrent cerebrovascular events. Axial brain MRI with fluid-attenuated inversion recovery (FLAIR) sequence showing an hyperintense lesion in the distribution of the left middle cerebral artery (**a**) without demonstrating restricted diffusion in the diffusion-weighted imaging sequences (**b**), compatible with the patient’s history of prior ischemic strokes. No new lesions were identified on the diffusion weighted imaging sequence of brain MRI confirming the diagnosis of a transient ischemic attack. Large intracranial vessels appeared normal on brain MRA (**c**). Anastomosis between the left ophthalmic artery and left middle meningeal artery was identified on thin Maximum Intensity Projection (MIP) MRA (circle; **d**). Meningeal branches in the area of craniocervical junction were dilated (circle; **e**). Significant dilatation of the branches of the internal carotid artery in the pterygoid canal was also visualized (circle; **f**)

**Fig. 2:**
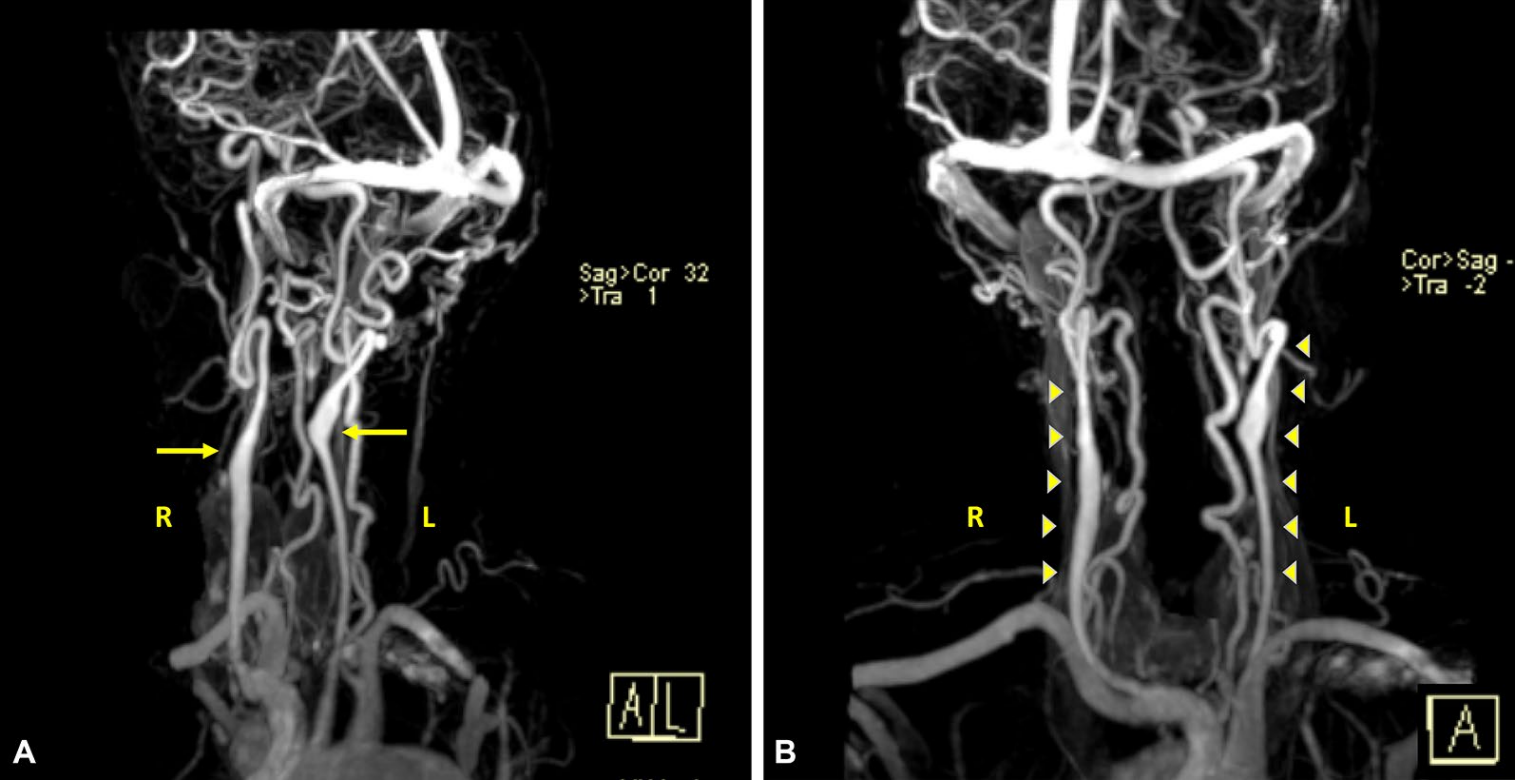
Neck MRA showing non-bifurcating carotid arteries. Anterolateral coronal view of neck MRA showing the common carotid arteries and the internal carotid arteries bilaterally originating from the carotid bulbs (arrows; **a**). Both vertebral arteries were visualized with high tortuosity. The common carotid arteries ascended in the neck as single carotid vessels without undergoing bifurcation and continuing as internal carotid arteries (arrowheads, **b**)

Brain MRI demonstrated a chronic ischemic lesion in the distribution of the left middle cerebral artery, compatible with the patient’s past medical history, without revealing any new ischemic lesions (Fig. 1a, b). Brain MRA revealed an extensive network of anastomotic arteries, while the circle of Willis appeared normal (Fig. 1c–f). Neuroimaging was completed with neck MRA demonstrating non-bifurcating carotid arteries bilaterally (Fig. 2). The common carotid arteries ascended in the neck as single carotid vessels without undergoing bifurcation and continuing as ICA. A digital subtraction angiography was suggested to the patient in order to better characterize the carotid anomaly and identify the branches that would normally originate from the ECA, but the patient refused.

Patient’s clinical examination was normal, without any signs of other developmental anomalies that could be associated with vessel abnormalities, as in the case of osteogenesis imperfecta. No headache following the patient’s symptoms or prior history of migraine were reported by the patient. Focal seizures were also included in the differential diagnosis. However, the patient’s history was negative for Jacksonian march or any positive symptoms such as paresthesia. Additionally, repeat electroencephalograms were normal.

Transient ischemic attack was considered the most probable diagnosis and complete work-up in order to identify the underlying mechanism was performed, including transthoracic and transesophageal echocardiography (revealing normal cardiac chambers’ size and further excluding right-to-left shunts), 24-h Holter rhythm monitoring, a-galactosidase levels, blood testing for autoimmune and hypercoagulable disorders; all diagnostic work-up was negative. Prolonged cardiac monitoring with implantable loop recorder was further suggested to the patient and long-term antiplatelet therapy and statins were administered as secondary stroke prevention therapy.

## Discussion

Embryologically, the ICA originates from the third aortic arch connecting to the dorsal aortic root. The ECA is developed by the connection of the ventral pharyngeal artery and the stapedial artery originating from the second aortic arch. Two prevailing hypotheses have been developed to explain the congenital anomaly of the non-bifurcating carotid artery.

The first hypothesis refers to the agenesis of the proximal segment of the ICA due to abnormal regression of the third aortic arch [2]. In that case, the common carotid artery continues as the ECA, which provides all the cervical branches as usual and later anastomoses with the second segment of ICA that continues intracranially. Characteristic findings in this hypothesis are the existence of an acute curve at the C1-C2 level indicating the anastomosis between ECA and ICA [3], the presence of an arterial stump at the site of the expected ICA origin [4], and the absence of a carotid bulb which is most characteristic for the first segment of the ICA [5].

The second hypothesis is that the ECA fails to develop, meaning that the common carotid artery directly continues as the ICA [6]. This hypothesis is supported by the finding of a similar diameter of the common carotid trunk compared to the healthy side with the presence of a carotid bulb [7].

In our patient, the carotid bulbs were well visualized bilaterally, indicating the successful development of the ICA. Regarding the association between this developmental anomaly and the patient’s recurrent cerebrovascular events, it remains unknown whether the finding of bilateral non-bifurcating carotid arteries was incidental, or if a true pathogenetic relationship actually existed. Previous reports underscore the presence of atherosclerosis in a single, non-bifurcating carotid vessel in symptomatic cases, postulating that shear stress, tortuosity or other local factors may lead to atheromatic plaque formation [8–10]. Despite that no atherosclerotic lesions were present in our case, one hypothesis could be that transient flow turbulence during hemodynamic changes through the single carotid vessels or through the associated extensive intracranial anastomosing arterial network may have partially contributed in the pathogenesis of the patient’s recurrent cerebrovascular events.

## Supplementary Information

**Additional file 1.** **Table S1** presenting the published cases with non-bifurcating carotid artery.

## Data Availability

All data are presented in the manuscript.

## Abbreviations

ICA: Internal carotid artery
ECA: External carotid artery
