# Supplementary material for: Bilateral non-bifurcating carotid arteries in a patient with recurrent cerebrovascular events
Source: Neurol Res Pract. 2021 Oct 18;3:55. doi: 10.1186/s42466-021-00154-9 (PMC8522077; doi:10.1186/s42466-021-00154-9)
Supplement: Supplementary file 1 — Additional file 1. Table S1 presenting the published cases with non-bifurcating carotid artery. [file 42466_2021_154_MOESM1_ESM.docx]

**Supplemental Table e-1:** Published cases with non-bifurcating carotid artery.

| **Author (Year)** | **Age, Sex** | **Side** | **Presence of**  **Atherosclerosis** | **Symptomatic**  **Cerebrovascular Disease** |
| --- | --- | --- | --- | --- |
| Basekim et al. (2004) [1] | 41, M | Bilateral | No | No |
| Franklin et al. (1988) [2] | Not Reported | Unilateral | Yes | Not Reported |
| Hu et al. (2018) [3] | 66, M | Right | No | No |
| Kaneko et al. (1996) [4] | 67, M | Left | Not Reported | Not Reported |
| Kim et al. (2015) [5] | 44, M | Right | No | Brief loss of consciousness |
|  | 60, M | Left | No | No |
| Kiyosue et al. (2009) [6] | 71, M | Right | No | No |
| Lambiase et al. (1991) [7] | 76, M | Left | Yes | No |
| Lourenco et al. (2014) [8] | 80, M | Left | No | No |
| Morimoto et al. (1990) [9] | 55, F | Left | Yes | No |
| Nakai et al. (2012) [10] | 71, M | Right | No | No |
| Nas et al. (2016) [11] | 42, F | Left | No | Confusion, dysarthria, and right hemiparesis |
| Ooigawa et al. (2006) [12] | 75, F | Left | No | No |
|  | 67, F | Left | No | No |
|  | 65, M | Left | No | No |
| Rodriguez et al. (2002) [13] | 66, M | Right | Yes | No |
| Sasaki et al. (2013) [14] | 68, M | Right | Yes | Amaurosis Fugax |
| Sase et al. (2020) [15] | 69, M | Right | Yes | No |
| Yoshida et al. (2016) [16] | 79, M | Left | Yes | Amaurosis Fugax |

**References used in the Supplement**

1. Basekim CC, Silit E, Mutlu H, Pekkafali MZ, Ozturk E, Kizilkaya E. Type I proatlantal artery with bilateral absence of the external carotid arteries. AJNR American journal of neuroradiology. 2004;25(9):1619-21.
2. Franklin PD, Lee RG, Allard JC, Gibbons GW, Costello P. Unilateral absence of the external carotid artery. Canadian Association of Radiologists journal = Journal l'Association canadienne des radiologistes. 1988;39(4):293-294.
3. Hu CC, Lai YJ, Lai WJ. Nonbifurcating Carotid Artery: A Case Report with a Review of Embryogenesis. The American journal of case reports. 2018;19:891-895.
4. Kaneko K, Akita M, Murata E, Imai M, Sowa K. Unilateral anomalous left common carotid artery; a case report. Annals of anatomy = Anatomischer Anzeiger : official organ of the Anatomische Gesellschaft. 1996;178(5):477-80.
5. Kim CH, Cho YD, Kang HS, Kim JE, Jung SC, Ahn JH, et al. Anomalous External Carotid Artery-Internal Carotid Artery Anastomosis in Two Patients with Proximal Internal Carotid Arterial Remnants. Korean journal of radiology. 2015;16(4):914-8.
6. Kiyosue H, Mori H, Tanoue S, et al. Non-bifurcating carotid artery coexisting with transverse sinus dural arteriovenous fistula. Neuroradiology. 2009;51(10):697-698.
7. Lambiase RE, Haas RA, Carney WI, Jr., Rogg J. Anomalous branching of the left common carotid artery with associated atherosclerotic changes: a case report. AJNR American journal of neuroradiology. 1991;12(1):187-9.
8. Lourenco P, Heran M. Asymptomatic non-bifurcating carotid artery. A case report and literature review. The neuroradiology journal. 2014;27(4):393-396.
9. Morimoto T, Nitta K, Kazekawa K, Hashizume K. The anomaly of a non-bifurcating cervical carotid artery. Case report. Journal of neurosurgery. 1990;72(1):130-132.
10. Nakai K, Kaji T, Uchino A, et al. Congenital external carotid-internal carotid artery anastomosis associated with contralateral non-bifurcating cervical carotid artery. Neuroradiology. 2012;54(5):521-523.
11. Nas OF, Karakullukcuoglu Z, Hakyemez B, Erdogan C. Dissection of a non-bifurcating cervical carotid artery. The neuroradiology journal. 2016;29(3):213-5.
12. Ooigawa H, Nawashiro H, Fukui S, Tsuzuki N, Katoh H, Kawaguchi T, et al. Non-bifurcating cervical carotid artery. Journal of clinical neuroscience : official journal of the Neurosurgical Society of Australasia. 2006;13(9):944-7.
13. Rodriguez HE, Ziauddin MF, Podbielski FJ, Durham JR, Clark ET. Congenital absence of the external carotid artery: atherosclerosis without a bifurcation. Journal of vascular surgery. 2002;35(3):573-5.
14. Sasaki T, Nagashima H, Oya F, Satoh D, Kobayashi S. Carotid artery stenting for atherosclerotic stenosis associated with non-bifurcating cervical carotid artery. Neurol Med Chir. 2013;53(4):228-32.
15. Sase T, Ito H, Uchida M, Tanaka Y. [Carotid Artery Stenting for Non-bifurcating Cervical Carotid Artery Stenosis:A Case Report]. No shinkei geka Neurological surgery. 2020;48(1):33-38.
16. Yoshida S, Ota T. Carotid Endarterectomy for Atherosclerotic Stenosis Associated with Non-bifurcating Cervical Carotid Artery: A Case Report. NMC case report journal. 2016;3(3):59-62.
